# Factors associated with work disability in employed cancer survivors at 24-month sick leave

**DOI:** 10.1186/1471-2407-14-236

**Published:** 2014-04-02

**Authors:** Peter van Muijen, Saskia FA Duijts, Karin Bonefaas-Groenewoud, Allard J van der Beek, Johannes R Anema

**Affiliations:** 1 Department of Public and Occupational Health, EMGO + Institute/VU University Medical Center, P.O. Box 7057, 1007 MB Amsterdam, The Netherlands; 2 Dutch Social Security Agency, Amsterdam, The Netherlands; 3 Research Center for Insurance Medicine, Amsterdam, The Netherlands

**Keywords:** Cancer, Survivors, Work disability, Return to work, Limitations, Sick leave

## Abstract

**Background:**

Identification of factors associated with work disability in cancer survivors on long term sick leave may support these survivors in choosing effective measures to facilitate vocational rehabilitation and return to work. Therefore, this study aims to disclose factors associated with work disability in cancer survivors at 24 months of sick leave.

**Methods:**

A cross sectional study was conducted. The study population consisted of employed sick-listed cancer survivors, aged between 18 and 64 years. They received a questionnaire at 24-month sick leave, the maximum period of sick leave allowed by Dutch social security legislation. Data were linked with the outcome of work disability assessment, as performed by the Dutch social security agency. A hierarchical multivariate logistic regression analysis was performed to identify factors associated with work disability.

**Results:**

Data of 351 valid cases were analysed. The multivariate analysis showed that, for cancer survivors at 24-month sick leave, Dutch nationality, higher education, receiving hormone therapy, metastatic disease, physical limitations and low self-reported work ability were associated with an increased risk for work disability.

**Conclusions:**

This study identified factors associated with work disability of employed cancer survivors at 24 months of sick leave. The results of the current study may serve as a starting point to investigate the course of work disability beyond the maximum period of 24 months of sick leave. In order to enhance work participation of cancer survivors beyond this term, prospective data on work disability in the Netherlands are required.

## Background

In the last decade, work participation of cancer survivors has received growing attention worldwide [1]. Previous studies have shown that cancer survivors are at risk for job loss, unemployment and work disability [2-9]. Cancer survivors who are unable to resume a former job not only face the risk of a financial loss [10]. That is, job loss can have a negative effect on recovery as well [11]. Return to work (RTW) may help cancer survivors to regain control, lead the way back to the former normal life, strengthen their self-confidence and support them to overcome negative side-effects of treatment [12,13]. Also, enhancement of work participation of cancer survivors is to the advantage of society at large, in reducing the costs of sick leave and work disability benefits, as well as productivity loss [14]. Therefore, the prevention of work disability in cancer survivors needs ongoing attention.

The impact of cancer and the potential side-effects of the treatment can lead to functional limitations, physical and/or psychological disabilities. This may create a barrier to work participation [15-17]. For instance, functional limitations leading to job changes or even exit from work were reported by 11% of breast cancer survivors in a study by Peuckmann et al. [18]. Further, in a cohort of male and female cancer survivors (mixed diagnoses), 27% of men and 32% of women reported work disabilities [7]. Also, in a cohort of cancer survivors (mixed diagnoses) with follow-up lasting between one to five years, 20% of respondents reported cancer-related disabilities and only half of those reporting disabilities were able to work [19]. Functional limitations and reduced work productivity can last up to several years after diagnosis, as reported by Yabroff. In this study, significant differences between cancer survivors and matched controls were found [20].

A number of factors negatively associated with work participation of cancer survivors have been identified. These factors are related to socio-demographics (e.g., old age, low education, low income), the disease (e.g., tumor site, chemotherapy, advanced tumor stage) and work-related characteristics (e.g., physical work demands) [1,15]. Still, the need to disclose unidentified factors associated with work participation of cancer survivors remains. That is, factors associated with work disability of cancer survivors present at a 24-month sick leave term, are poorly investigated. Most studies focus on RTW, are either based on hospital data or data of an occupational health service and relate to a period of absence from work of three up to 18 months [21,22]. During this period, relatively shortly after diagnosis, potential long-term sequelae of cancer and cancer treatment, possibly associated with work disability, may not be apparent.

Recently, a number of factors associated with work disability assessment outcomes were identified. That is, in a prospective study it was reported that at 10-month sick leave, perception of health care providers on cancer survivors’ work ability and experienced influence on RTW, both reported by workers, were significantly associated with the level of work disability at 24 months [23]. As factors at 10-month sick leave exert an influence on work disability at 24 months, this questions what factors are associated with sustained work disability as assessed at 24-month sick leave and beyond. Identification of barriers of work participation, i.e., factors associated with work disability at specific points in time, can make it possible to offer the adequate support, using resources in an optimal way. Therefore, this study aims to identify factors associated with the level of work disability at 24-month sick leave in cancer survivors. Herewith, the level of work disability is defined as wage loss related to functional limitations, which is present practice in the Dutch social security system.

## Methods

### Design

A cross-sectional design was used for the current study, for which two data sources were used: 1) questionnaire data, and 2) register data of work disability assessments. The period of inclusion started in July 2011 and ended in February 2012. Data were collected when study participants approached the maximum term of 24-month sick leave and applied for a work disability benefit at the Dutch Social Security Agency (SSA). In the Netherlands, the SSA is responsible for the assessment of work disability of workers on long-term sick leave. The assessment of functional abilities at 24-month sick leave (the maximum period allowed by law) is done by an insurance physician. If applicable, based on the physician’s report, a labour expert calculates the loss of former wages earned. In 2009, 65% of Dutch cancer survivors who applied for a work disability benefit was granted a full work disability benefit [24]. This implies a wage loss of ≥ 80% of former wages earned.

Questionnaires were sent to the participants at their home address. Upon receipt, data of the questionnaires were linked to SSA data. The study was approved by the Medical Ethics Committee of the VU University Medical Center.

### Study population

The study population consisted of sick-listed employed workers (hereafter designated as workers) who were registered at the SSA. They were aged between 18 and 64 years. All workers had a reported diagnosis of cancer, and were approaching a sick leave term of 24 months. Diagnosis had to be confirmed within the first six months of sick leave. Workers were excluded if they received active chemotherapy and/or radiotherapy treatment, if they had a previous diagnosis of cancer but applied for a work disability benefit due to another somatic or psychiatric disorder, if they were self-employed, if they were applying for a revision of a previous work disability assessment, or if they were employed in a so-called sheltered workplace.

### Study procedure

Potentially eligible participants of our study were selected at the head office of the SSA. During the period of inclusion, the list of new work disability benefit applications was checked by one author (KBG or PvM) every week. Based on this list of social security numbers and corresponding documents, we selected the sick-listed workers with a diagnosis of cancer, as reported in the attached medical records. After starting the selection, in case of doubt, cases were included based on consensus. Potentially eligible participants received a questionnaire, an informed consent form, and information stating the aim and background of the study. A postage-paid return envelope (to the Research Center for Insurance Medicine at the EMGO + Institute at the VU University Medical Center) and an introductory letter, by the chief medical officer of the SSA, were added. This letter stated the independency of the researchers and stressed that participation would be of no influence on the outcome of the work disability assessment. Participants had to complete the informed consent form by hand and affix a signature. On receipt of the signed informed consent form and the questionnaire, we linked the latter with personal data (i.e., family name, address, birth date), as collected at the SSA head office, and entered these data in a secured database. The chief medical officer of the SSA gave permission to access the SSA’s registry data. A reminder was sent after two weeks. Also, a reminder was sent in case of a missing signature on the informed consent form. Questionnaires of respondents lacking a completed form were destroyed. All respondents received a gift voucher.

Workers who reported to receive chemotherapy and/or radiotherapy and workers of whom the main reason for application was not cancer-related, were excluded. They were sent a letter explaining the reason of exclusion. The questionnaires were checked for completeness and, if necessary, respondents were contacted to supply missing data.

### Variables

The independent and dependent variables were collected through questionnaires as used in earlier studies on cancer survivorship and return to work [1,11,25-27].

### Independent variables

#### ***Socio-demographics***

The following socio-demographic characteristics were determined: (a) age (in years), (b) gender (male; female), (c) marital status (single; married/living with partner; divorced/widowed), (d) number of children, (e) principal wage earner (yes; no), (f) educational level (no education/primary school/lower vocational education; secondary school; vocational education/upper secondary school; upper vocational education/university), (g) nationality (Dutch; non-Dutch).

### Health determinants

The following health characteristics were assessed: (a) tumor type, (b) extensive disease (negative lymph nodes; positive lymph nodes; metastasis), (c) treatment modalities (surgery; radiotherapy; chemotherapy; hormone therapy; bone marrow transplant; immunotherapy), (d) being free of disease (yes; no; don’t know), (e) comorbidity (number of additional diseases). Physical symptom burden was measured using (f) the physical dimension score of the Sickness Impact Profile (SIP), covering three scales, i.e., Body Care and Movement, Ambulation, and Mobility [28]. Also, (g) fatigue, (h) depressive mood, and (i) global health were measured using the Functional Assessment of Chronic Illness Therapy-Fatigue Scale (FACIT-F) [29], the Center for Epidemiologic Studies Depression Scale (CES-D) [30], and the European Organisation for Research and Treatment of Cancer Quality of Life Questionnaire-C30 (EORTC-QLQ-C30) [31], respectively.

### Work-related determinants

The following characteristics of the previous job held were determined: (a) type of job (white collar; civil servant; blue collar; health care worker), (b) job tenure (in years), (c) working hours (hours/week), (d) shift work (yes; no), (e) managerial tasks (yes; no), (f) number of supervised co-workers, (g) work demands (psychological; physical; both), (h) company size (number of employees), and (i) work ability expectations (same; increase; decrease; don’t know). Related to the present (j) work status (working; not working), (k) the actual number of working hours were determined. Finally, with the first three items of the Work Ability Index (WAI) [1] current work ability compared to life time best, (m) current work ability related to physical work demands and (n) current work ability related to psychological work demands, were measured [32].

### Dependent variable

The primary outcome variable was the level of work disability after 24 months of sick leave. This was operationalised by dichotomising the results of the work disability assessments, the entitlement for work disability compensation, as performed by the SSA. In the Netherlands, the level of work disability is assigned to one out of four categories, depending on wage loss or sustainable absence of functional abilities. If functional abilities are assessed present, wage loss can be either (1) less than 35%, (2) in between 35 to 80%, or (3) over 80% of former wages earned. The compensation granted can be none, partial, or complete, respectively. If a person has no labour capacities (sustainable absence of functional abilities) the claimant is granted (4) a compensation by the Benefit Act for the fully and sustained work disabled. The participants with a wage loss of less than 80% were grouped together, as well as those with a wage loss equal to or more than 80% and those with a permanent and sustainable work disability. Herewith, workers assessed as still being able to earn an income were distinguished from those unable to earn an income, i.e., incomplete versus complete work disability.

### Statistical analysis

The following variables were binominal: gender, nationality, work status, principal wage earner, shift work, managerial tasks, and treatment modalities. A number of variables was dichotomized: age, job tenure, working hours per week in previous job, the number of supervised co-workers, working hours per week in present job, scores of the SIP, FACIT-F, EORTC-QLQ-C30, and WAI, using the median as a cut-off point. For the CES-D, the variable was dichotomized at a score of 16, the predetermined cut-off point most often used for likely cases of clinical depression [30]. Categorical variables were marital status, number of children, education, type of job, work demands, company size, comorbidity, tumor type, extensive disease, and being free of disease.

The association between independent variables and the binominal level of work disability at 24 months (wage loss <80%; ≥80%) were analysed with univariate and multivariate methods. For univariate analysis, a Chi-square test was performed using a cut-off for p-values of 0.20. The remaining significant independent variables of univariate analysis were then tested for multicollinearity and accepted in a logistic regression model if correlation coefficients were ≥ -0.6 and ≤0.6 [33]. Next, for each category of variables, i.e., socio-demographics, work-related characteristics and health characteristics, multiple logistic regression analysis was performed, using a backward stepwise method. For each category of variables, this resulted in a logistic regression model presenting variables associated with work disability at 24 months of sick leave. Next, using the results of these three backward stepwise models a final model was built. In this final model, variables of the three categories i.e. socio-demographics, health determinants and work-related determinants were added in consecutive order. This resulted in a final model, presenting variables associated with work disability at 24 months of sick leave, controlling in a hierarchical way for socio-demographics, health determinants and work-related determinants. The association for each independent variable and the level of work disability at 24 months was calculated using odds ratios (OR). In the logistic regression analyses, a cut-off for p-values of 0.1 (Wald statistics) for independent variables was chosen. The Hosmer-Lemeshow test was used to assess the goodness of fit. All analyses were performed using SPSS 20 [34].

## Results

### Characteristics of the study population

During the period of inclusion, 13,023 employed workers applied for a work disability benefit. Of these 1,307 had a diagnosis of cancer of whom 995 met the inclusion criteria. These 995 workers were sent a questionnaire, of whom 528 responded. Based on exclusion criteria (i.e., data retrieved from received questionnaires) and based on supplementary SSA data, 136 of the respondents were excluded. Finally, 392 cancer survivors were included. In 41 of the 392 cancer survivors, the level of work disability could not be retrieved, leaving 351 valid cases. The mean age of these respondents was 51.1 years (SD 7.4 years) and 36% were men. The majority (79%) was in a relationship and 27% was educated at high vocational or university level. Related to the category health determinants, more specifically tumor type, breast cancer was reported in 40%, haematological cancer in 14%, and cancer of the digestive system in 13% of the cases. Other tumor types were reported in 33% of cancer survivors. Related to extensiveness of disease, 52% of respondents reported having negative lymph nodes and 42% reported being free of disease. In the category work-related determinants, 40% of respondents worked in a blue collar job and 29% in a white collar job. A total of 60% of the respondents reported their company to have > 100 employees, and 40% reported to be actually working in paid labour. Positive work ability expectations were reported by 35% respondents, and 17% expected work ability to stay the same.

### Level of work disability

Regarding the dependent variable, we found that of the 351 cancer survivors, 92 had less than 35%, 101 between 35 and 80%, and 97 over 80% loss of former wages earned, as assessed by the SSA at 24-month sick leave. In 61 of the cancer survivors, no labour capacities (full and sustained work disability) were present.

### Cancer survivors and determinants of work disability

Results of the univariate analyses, in which the relationship between the independent variables and the level of work disability at 24-month sick leave were tested, are presented in Tables 1 and 2. In the multivariate analyses, all variables that showed a p-value of <0.2 in the univariate analyses (n = 21) were used to build a final multivariate model. The backward stepwise analyses showed associations at a level of p < 0.1 for nationality, education, extensive disease, hormone therapy, being free of disease, the physical dimension score (SIP), global health (EORTC-QLQ-C30 score), work demands, and current work ability compared to life time best score (WAI). These nine variables were entered in the hierarchical model in consecutive order as listed above. The last (ninth) step of the hierarchical multivariate analyses is presented in Table 3. The Hosmer-Lemeshow test revealed that this model had a good fit (p = 0.948). In this final model, we found associations at a level of p < 0.1 for nationality, education, hormone therapy, extensive disease, the physical dimension score (SIP), and current work ability compared to life time best score (WAI). Specifically, cancer survivors of non-Dutch nationality were less at risk for work disability than their Dutch counterparts (OR 0.15; CI 0.02-0.95); an education at the level of secondary school (OR 4.80; CI 1.72-13.42) and vocational education/upper secondary school (OR 2.78; CI 1.16-6.69) were both associated with an increased risk for work disability when compared to the lowest educational category. Further, receiving hormone therapy (OR 2.20; CI 1.08-4.47), having metastatic disease (OR 4.51; CI 1.65-12.34) and reporting a high level of physical complaints (SIP) (OR 2.62; CI 1.34-5.14) were all associated with an increased risk for work disability. A high score on current work ability compared to life time best (WAI) (OR 0.09; CI 0.04-0.19) was associated with a decreased risk for work disability.

**Table 1:** Characteristics of employed cancer survivors*

**Socio-demographics**	**Categories**	**n (%)**	**Disability (<80%; ≥80%)**	**p-value**
			**n (%); n (%)**	
Gender	Male	125 (36)	68 (54); 57 (46)	0.870
	Female	226 (64)	125 (55); 101 (45)	
Age in years	< 52	163 (46)	98 (60); 65 (40)	0.072
	≥ 52	188 (54)	95 (50); 93 (50)	
Marital status	Single	35 (10)	22 (63); 13 (37)	0.413
	Married/living with partner	278 (79)	153 (55); 125 (45)	
	Divorced/widowed	38 (11)	18 (47); 20 (53)	
No. of children	None	90 (26)	55 (61); 35 (39)	0.576
	1	46 (13)	24 (52); 22 (48)	
	2	139 (40)	75 (54); 64 (46)	
	> 2	76 (21)	39 (51); 37 (49)	
Principal wage earner	Yes	193 (55)	109 (56); 84 (44)	0.465
	No	156 (45)	82 (53); 74 (47)	
Nationality	Dutch	340 (97)	184 (54); 156 (46)	0.069
	Non-Dutch	11 (3)	9 (82); 2 (18)	
Education	None/ primary/lower vocational	91 (26)	49 (54); 42 (46)	0.102
	Secondary school	60 (17)	25 (42); 35 (58)	
	Vocat. education/upper sec. school	105 (30)	63 (60); 42 (40)	
	Upper vocat. education/university	94 (27)	56 (60); 38 (40)	
**Health determinants**				
Tumor type	Mamma	143 (40)	93 (65); 50 (35)	0.024
	Urinary tract	20 (6)	11 (55); 9 (45)	
	Urogenital (m)	11 (3)	5 (46); 6 (54)	
	Urogenital (f)	17 (5)	10 (59); 7 (41)	
	Respiratory tract	24 (7)	6 (25); 18 (75)	
	Digestive system	45 (13)	21 (47); 24 (53)	
	Head and neck	18 (5)	12 (67); 6 (33)	
	Haematological	50 (14)	25 (50); 25 (50)	
	Central nerve system	10 (3)	4 (40); 6 (60)	
	Other	13 (4)	6 (42); 7 (58)	
Extensive disease	Negative lymph nodes	180 (52)	103 (57); 77 (43)	0.001
	Positive lymph nodes	116 (34)	72 (62); 44 (38)	
	Metastasis	50 (14)	16 (32); 34 (68)	
Treatment modalities	Surgery yes	242 (69)	141 (58); 101 (42)	0.066
	no	109 (31)	52 (48); 57 (52)	
	Radiotherapy yes	210 (60)	114 (54); 96 (46)	0.748
	no	141 (40)	79 (56); 62 (44)	
	Chemotherapy yes	253 (72)	134 (53); 119 (47)	0.221
	no	98 (28)	59 (60); 39 (40)	
	Hormone therapy yes	107 (30)	69 (65); 38 (35)	0.018
	no	244 (70)	124 (51); 120 (49)	
	Bone marrow transplant yes	19 (6)	6 (32); 13 (68)	0.035
	no	332 (94)	187 (56); 145 (44)	
	Immunotherapy yes	31 (9)	18 (58); 13 (42)	0.718
	no	320 (91)	175 (55); 145 (45)	
Being free of disease	Yes	147 (42)	99 (67); 48 (33)	0.000
	No	106 ( 31)	42 (40); 64 (60)	
	Don’t know	94 (27)	48 (51); 46 (49)	
Comorbidity	No. of additional diseases 0	195 (56)	112 (58); 83 (52)	0.324
	No. of additional diseases 1	51 (14)	31 (61); 20 (39)	
	No. of additional diseases 2	52 (15)	25 (48); 27 (52)	
	No. of additional diseases ≥ 3	53 (15)	25 (47); 28 (53)	
**Work-related determinants**				
Type of job	White collar	102 (29)	59 (58); 43 (52)	0.459
	Civil servant	33 (9)	15 (45); 18 (55)	
	Blue collar	139 (40)	73 (53); 66 (47)	
	Health care worker	77 (22)	46 (60); 31 (40)	
Job tenure in years	≤ 10	173 (49)	97 (56); 76 (44)	0.687
	> 10	178 (51)	96 (54); 82 (46)	
Working hours a week	≤ 32	177 (50)	90 (51); 87 (49)	0.116
	> 32	174 (50)	103 (59); 71 (41)	
Shift work	Yes	115 (33)	67 (58); 48 (42)	0.371
	No	235 (67)	125 (53); 110 (47)	
Managerial tasks	Yes	70 (20)	41 (59); 29 (41)	0.488
	No	278 (80)	150 (54); 128 (46)	
No. of supervised co-workers	≤ 7	35 (50)	18 (51); 17 (49)	0.225
	> 7	35 (50)	23 (66); 12 (34)	
Work demands	Psychological and physical	168 (48)	93 (55); 75 (45)	0.084
	Psychological	106 (30)	65 (61); 41 (39)	
	Physical	76 (22)	34 (45); 42 (55)	
Company size	No. of employees 1-9	37 (10)	21 (57); 16 (43)	0.379
	No. of employees 10-99	104 (30)	51 (49); 53 (51)	
	No. of employees ≥ 100	208 (60)	119 (57); 89 (43)	
Work ability expectations	Same	40 (17)	36 (90); 4 (10)	0.000
	Increase	79 (35)	64 (81); 15 (19)	
	Decrease	31 (13)	15 (48); 16 (52)	
	Don’t know	81 (35)	58 (72); 23 (28)	
Work status	Working	141 (40)	122 (87); 19 (13)	0.000
	Not working	210 (60)	71 (34); 139 (66)	
Actual working hours per week	≤ 20	79 (56)	64 (81); 15 (19)	0.030
	> 20	62 (44)	58 (94); 4 (6)	

*Due to missing data n varies (range: 70–351); p-value: result of Chi-square test, univariate associations between independent variables and work disability.

**Table 2:** Questionnaire scores of employed cancer survivors

**Variables**	Cut-off value*	**n (%)**	**Disability (<80%; ≥ 80%)**	**p-value†**
			**n (%); n (%)**	
Physical dimension score (SIP; 0–100)	≤ 3.77‡	179 (51)	129 (72); 50 (28)	0.000
	> 3.77	172 (49)	64 (37); 108 (63)	
Fatigue (FACIT-F; 0–52)	≤ 27§	173 (49)	73 (42); 100 (58)	0.000
	> 27	178 (51)	120 (67); 58 (33)	
Depressive mood (CES-D; 0–60)	≤ 16∥	188 (54)	114 (61); 74 (39)	0.017
	> 16	163 (46)	79 (49); 84 (51)	
Global health (EORTC-QLQ-C30; 0–100)	≤ 58.33¶	160 (46)	61 (38); 99 (62)	0.000
	> 58.33	191 (54)	132 (69); 59 (31)	
Current work ability (WAI; 0–10)	≤ 4**	143 (48)	52 (36); 91 (64)	0.000
	> 4	158 (52)	131 (83); 27 (17)	
Physical demands (WAI; 0–5)	≤ 3††	142 (62)	97 (68); 45 (32)	0.003
	> 3	86 (38)	74 (86); 12 (14)	
Psychological demands (WAI; 0–5)	≤ 3‡‡	121 (52)	83 (69); 38 (31)	0.021
	> 3	110 (48)	90 (82); 20 (18)	

*Cut-off value = median (except for CES-D; the predetermined cut-off point is used here); †Result of Chi-square test; Range as reported by participants: ‡0-79.83; §0-52; ∥0-57; ¶0-100; **0-10; ††1-5; ‡‡1-5.

**Table 3:** Multivariate associations between independent variables and work disability in employed cancer survivors

Socio-demographics*	**Categories**	**Odds ratio (95% CI)**	**p-value**
Nationality	Dutch; non-Dutch	0.147 (0.02-0.95)	0.044
Education			0.015
	None/primary/lower vocational education	Ref.	
	Secondary school	4.80 (1.72-13.42)	0.003
	Vocational education/upper sec. school	2.78 (1.16-6.69)	0.022
	Upper vocational education/university	1.68 (0.65-4.38)	0.286
Health determinants*			
Being free of disease			0.358
	Yes	Ref.	
	No	1.44 (0.64-3.23)	0.380
	Don’t know	1.69 (0.80-3.57)	0.166
Hormone therapy	No; yes	2.20 (1.08-4.47)	0.029
Extensive disease			0.013
	Negative lymph nodes	Ref.	
	Positive lymph nodes	1.23 (0.60-2.52)	0.582
	Metastasis	4.51 (1.65-12.34)	0.003
Physical dimension score (SIP; 0–100)	≤ 3.77; > 3.77†	2.62 (1.34-5.14)	0.005
Global health (EORTC-QLQ-C30; 0–100)	≤ 58.33; > 58.33‡	0.83 (0.40-1.70)	0.607
Work-related determinants*			
Work demands			0.109
	Psychological and physical	Ref.	
	Psychological	1.04 (0.50-2.19)	0.913
	Physical	2.51 (1.05-6.01)	0.039
Current work ability (WAI; 0–10)	≤ 4; > 4§	0.09 (0.04-0.19)	0.000

*For binairy variables the reference value is listed first; Range as reported by participants: †0–79.83; ‡0–100; §0–10.

## Discussion

### Main findings

The aim of this study was to identify determinants associated with work disability defined as wage loss related to functional limitations, at 24-month sick leave in cancer survivors. For cancer survivors at 24 months of sick leave, Dutch nationality, higher education, hormone therapy, metastatic disease, a high physical dimension score (SIP) and low current work ability, compared to life time best score (WAI), were associated with an increased risk for work disability.

### Interpretation of the findings and comparison with other studies

In this study, we found that higher education (at the level of secondary school and vocational education/upper secondary school) was associated with an increased risk for work disability. This result differs from previous studies, possibly due to the specific legislation applied in the Netherlands, in which work disability not only relates to limitations and loss of functional abilities, but to wage loss as well. As a consequence, if less paid jobs are associated with a low educational level, then it is likely that on assessment of work disability, a low educated cancer survivor suffers only little wage loss. That is, a low educated cancer survivor still able to work and earn a major part of the previous income is less likely to be granted a disability benefit. The mechanism involved could also relate to the presence of disease induced disabilities and limitations, making it harder to meet the cognitive job demands of the better educated white collar workers. As a consequence, higher educated white collar workers face wage loss, as only less complex and consequently less paid jobs meet their remaining abilities. The suggested mechanism mentioned above agrees with the findings of previous studies, which have reported long-term negative effects of diagnosis and treatment on the ability to memorize, concentrate, direct attention and solve problems [35-37]. The results of previous studies indicate that in cancer survivors a low educational level is negatively associated with work participation. This in turn calls for a policy in order to support these workers in their vocational rehabilitation.

We also found that workers of non-Dutch nationality had a decreased risk for work disability. This finding must be interpreted with caution and may be due to coincidence, considering the small number of these workers in our study (n = 11).

Also, we found that receiving hormone therapy was associated with an increased risk for work disability. In breast cancer survivors, this could be due to the occurrence of treatment induced menopausal symptoms, resulting from hormone therapy, that may have a negative impact on cognitive tasks and/or social and emotional aspects of work ability [38]. Our findings indicate that side effects of hormone therapy should not be underestimated and suggest that risk for work disability at 24-month sick leave may be reduced if at the start of hormone therapy attention is given to possible side effects of treatment. That is, job and workplace accommodations and offering alternative tasks may support those suffering from side effects of hormone therapy and may facilitate work participation.

Cancer survivors with metastatic disease had an increased risk for work disability. This finding concurs with previous studies that describe the negative relationship between the extensiveness/burden of disease and work ability [39-42]. Metastatic disease and a related poor health condition, due to symptoms such as fatigue, combined with negative side-effects of ongoing treatment may limit functional abilities. Symptoms associated with metastatic disease may add up to such an extent that even activities of daily living are difficult to meet and work participation is not possible [43].

Related to the SIP, we found that as the number of limitations in the physical domain of the SIP increases, the risk for work disability increases as well. Our finding concurs with the results of a previous study on cancer survivors (mixed diagnoses) and work disability, which indicated that survivors were leaving the labour force or were functioning less fully at work than before becoming ill [44]. In this study, the strongest predictors of work disability were physical dysfunction, measured by the SIP, and disease stage.

Finally, in our sample of cancer survivors, a high score on work ability (WAI current) was associated with a reduced risk for work disability. This finding agrees with the results of a previous study in which work ability assessed at six months sick leave (using the WAI) strongly predicted RTW at 18 months [25]. Therefore, considering our findings, it is possible that during sustained sick leave, measurement of self-assessed work ability at fixed intervals may also be helpful to identify cancer survivors at risk for work disability after a period of 24-month sick leave.

### Strengths and limitations

A strength of our study is that the sample was drawn from the entire Dutch working population. Another strength is that the primary outcome of the study was not self reported but based on the assessment by an independent insurance physician and a labour expert, following uniform guidelines, based on national legislation being practised at all SSA offices nationwide. This warrants a uniform procedure by which work disability is judged. However, in assessing functional abilities of workers, insurance physicians use a standardized List of Functional Abilities (LFA), which is a non-validated instrument [45]. Also, a previous study on work disability assessments found small to moderate systematic variations in the outcome of work disability assessments related to inter-doctor variations, which can be considered a limitation of the present study [46]. Another limitation of the study is its cross-sectional design, which makes it impossible to disclose causal relationships. Also, the study results relate to Dutch social security legislation, in which functional limitations and wage loss define the level of work disability. This impedes the generalisation to workers in other countries.

### Practical implications

Work participation of cancer survivors may be enhanced if factors hindering this process are identified and open to change or otherwise given attention in a supportive way. This study identified six factors associated with work disability of employed cancer survivors at 24-month sick leave. The association of nationality with work disability needs further clarification, considering the small number of respondents in our study of non-Dutch nationality. For future studies, a policy to sample a sufficient number of workers of non-Dutch nationality is advised. The level of former education may also help to identify sick listed workers at risk for work disability and, though educational level may not be changed easily, vocational training and courses that focus on acquiring new skills may support sick listed cancer survivors and enhance their work participation. Caregivers involved in vocational rehabilitation must be aware of possible long-term impact of hormone therapy on work disability, encourage cancer survivors to reveal and discuss possible side effects of hormone therapy and advise measures to cope with these, preferably at the start of therapy. The presence of metastatic disease is a factor unlikely to change, but to caregivers involved, this aspect may serve as a warning sign and draw attention to individuals at risk for work disability. Likewise, monitoring of physical limitations during prolonged sick leave may help to identify those at risk for work disability. In cancer survivors apt to rehabilitation, these limitations could possibly diminish with the use of tailored interventions that may reduce the risk for work disability. The data suggest that, considering the results of a previous study on repeated work ability scores [25], monitoring self-assessed work ability scores during sustained sick leave, may support the identification of cancer survivors at risk for work disability at 24-month sick leave. For a part, our results may also apply to cancer survivors abroad. Therefore, in the European context, further research on long term effects of hormone therapy, the survey of physical limitations and use of self-assessed work ability in identifying cancer survivors at risk for work disability, is suggested.

## Conclusions

The results of the current study may serve as a starting point to investigate the course of work disability beyond the 24-month sick leave term. In order to enhance work participation of cancer survivors beyond this term, prospective data on work disability are required and called for.

## Competing interests

PvM is employed by the Dutch Social Security Agency. For the remaining authors, no conflicts of interest were declared.

## Authors’ contributions

All authors were involved in designing the study. KBG and PvM collected and analysed the data. All authors reviewed the data and were involved in final analysis and conclusions. PvM and SD wrote the first draft of the manuscript to which all authors subsequently contributed. All authors read and approved the final manuscript.

## Pre-publication history

The pre-publication history for this paper can be accessed here:

http://www.biomedcentral.com/1471-2407/14/236/prepub
